# Probiotics for the Treatment of Overweight and Obesity in Humans—A Review of Clinical Trials

**DOI:** 10.3390/microorganisms8081148

**Published:** 2020-07-29

**Authors:** Michał Wiciński, Jakub Gębalski, Jakub Gołębiewski, Bartosz Malinowski

**Affiliations:** Department of Pharmacology and Therapeutic, Faculty of Medicine, Collegium Medicum in Bydgoszcz, Nicolaus Copernicus University, 85-090 Bydgoszcz, Poland; wicinski4@wp.pl (M.W.); jakub.golebiewski93@gmail.com (J.G.); bartosz.malinowski@cm.umk.pl (B.M.)

**Keywords:** obesity, overweight, gut microbiota, probiotic, *Bifidobacterium*, *Lactobacillus*

## Abstract

The World Health Organization (WHO) reports that 400 million people are obese, and over 1.6 billion adults are overweight worldwide. Annually, over 2.8 million people die from obesity-related diseases. The incidence of overweight and obesity is steadily increasing, and this phenomenon is referred to as a 21st-century pandemic. The main reason for this phenomenon is an easy access to high-energy, processed foods, and a low-activity lifestyle. These changes lead to an energy imbalance and, as a consequence, to the development of body fat. Weight gain contributes to the development of heart diseases, skeletal system disorders, metabolic disorders such as diabetes, and certain types of cancer. In recent years, there have been many works linking obesity with intestinal microbiota. Experiments on germ-free animals (GFs) have provided much evidence for the contribution of bacteria to obesity. The composition of the gut microbiota (GM) changes in obese people. These changes affect the degree of energy obtained from food, the composition and secretory functions of adipose tissue, carbohydrate, and lipid metabolism in the liver, and the activity of centers in the brain. The study aimed to present the current state of knowledge about the role of intestinal microbiota in the development of obesity and the impact of supplementation with probiotic bacteria on the health of overweight and obese patients.

## 1. Introduction

In 2016, over 1.9 billion adults were overweight. There were over 650 million obese people in this group. Most people live in countries where overweight and obesity kill more people than underweight. According to WHO, around 2.8 million people die annually due to overweight or obesity. Abnormal or excessive fat accumulation is genetic, environmental, metabolic, and psychological. The leading cause of obesity and overweight is the lack of energy balance between the number of calories supplied and burned. There are two types of obesity: primary and secondary obesity. Primary obesity is mainly associated with environmental factors such as physical inactivity and an improper diet. Secondary obesity is caused by comorbidities: polycystic ovary syndrome, hypothyroidism, and genetic diseases. Obesity and overweight can cause many diseases, such as cardiovascular disease (atherosclerosis and hypertension), type II diabetes, osteoarthritis, and some cancers. Obesity and overweight, due to the speed of development, have become an epidemic of the 21st century. Research is underway around the world to better understand the etiology and pathogenesis of obesity. In recent years there have been articles showing the potential relationship between changes in the composition of intestinal microbiota and obesity [1,2,3]. Therefore, in this review, we will discuss the role of gut microbiota (GM) in the formation and development of obesity. We will present mechanisms explaining the role of intestinal microbiota in obesity formation. Additionally, we will discuss the potential use of probiotics in the treatment of overweight or obesity using data from clinical trials in humans. Finally, we will propose future research and methodological approaches to understand better the interactions between intestinal microbiota and obesity.

## 2. Methods

### 2.1. Data Sources and Searches

We conducted a qualitative systematic review of randomized clinical trials (RCTs), published in English, for overweight and obese people. The MEDLINE, PubMed, and EMBASE databases were used for the literature search. Publications were searched between January 2010 and December 2019. We used the PICO scheme as a literature search strategy. P (patient) [overweight/obesity/adiposity]; I (intervention) [*Lactobacillus*/*Bifidobacterium*/probiotics]; C (not applied); and O (outcome) [weight loss/fatty tissue reduction/thighs circumference reduction/waist circumference reduction]. The search terms included “probiotics”, “*Bifidobacterium*”, “*Lactobacillus*”, “obesity”, “overweight”, “adipose tissue”, “waist circumference”, “weight reduction”, and “thigh circumference”. The search equation was defined following the formulation [probiotics OR Lactobacillus OR Bifidobacterium] AND [obesity OR overweight] AND [weight loss OR waist circumference reduction OR thigh circumference reduction OR fat reduction].

### 2.2. Eligibility Criteria

The inclusion criteria: (1) randomized controlled trial (either parallel groups or cross-over); (2) clinical trials in humans (pregnant women, infants, children, teenagers, adults, and the elderly); (3) overweight or obese persons with/without diabetes, metabolic syndrome, cardiovascular diseases, and non-alcoholic steatosis; (4) studies in English; (5) the effects of probiotics and/or synbiotics on metabolic or anthropometric indicators have been determined; (6) sufficient information on anthropometric and metabolic indicators has been provided in both the study and control groups; and (7) probiotics and/or synbiotics have been administered for at least 3 weeks. 

Exclusion criteria: (1) studies other than RCT; (2) interventions with probiotics/synbiotics/prebiotics without a suitable placebo group; (3) studies in which probiotic bacterial species are not clearly identified have been excluded; (4) key data are incomplete; (5) studies that were case studies, reviews, letters, or conference abstracts; (6) antibiotic intake during the 1 month prior to the study; (7) use of products enriched with prebiotics and probiotics (for at least 4 weeks prior to the control visit) and products with high dietary fiber content; (8) use of obesity drugs during the last 3 months; (9) use of glucocorticosteroids, non-steroidal anti-inflammatory drugs, proton pump inhibitors, and H2 receptor inhibitors; (10) cancer or chemotherapy/radiotherapy; (11) nicotine, alcohol, or drug abuse; (12) autoimmune diseases, gastrointestinal diseases and allergic diseases (atopic eczema, allergic rhinitis, or asthma); (13) persons in whom weight loss is contraindicated; and (14) irregular lifestyles. 

In addition, the exclusion criterion in studies involving people with non-alcoholic fatty liver disease (NAFLD) was a chronic liver disease caused by Wilson’s disease, autoimmune hepatitis, hepatitis B and C, HIV, and a-1-antitrypsin deficiency. In studies involving pregnant women, it was excluded: (1) women under 18 years of age; (2) multiple pregnancies; (3) fetal anomalies; and (4) previously diagnosed diabetes. The exclusion criteria in studies with infants were: (1) malformation and (2) caesarean delivery.

### 2.3. Quality Assessment

The risk of bias was independently assessed by two researchers (J.Go and B.M) according to the criteria detailed in the Cochrane Collaboration tool [4]. Selected publications were evaluated using the following criteria: random sequence generation, allocation concealment, blinding of participants and personnel, blinding of outcome assessment, incomplete outcome data, selective reporting, and other bias. The risk of bias in each field was classified as low, high, or unclear. In order to minimize or avoid errors in the results, the included studies have been grouped by population (pregnant women, infants, children, teenagers, adults, and the elderly). Studies of poor quality were not included if they affected the final results.

## 3. Results

### 3.1. Search Results

**Figure 1:**
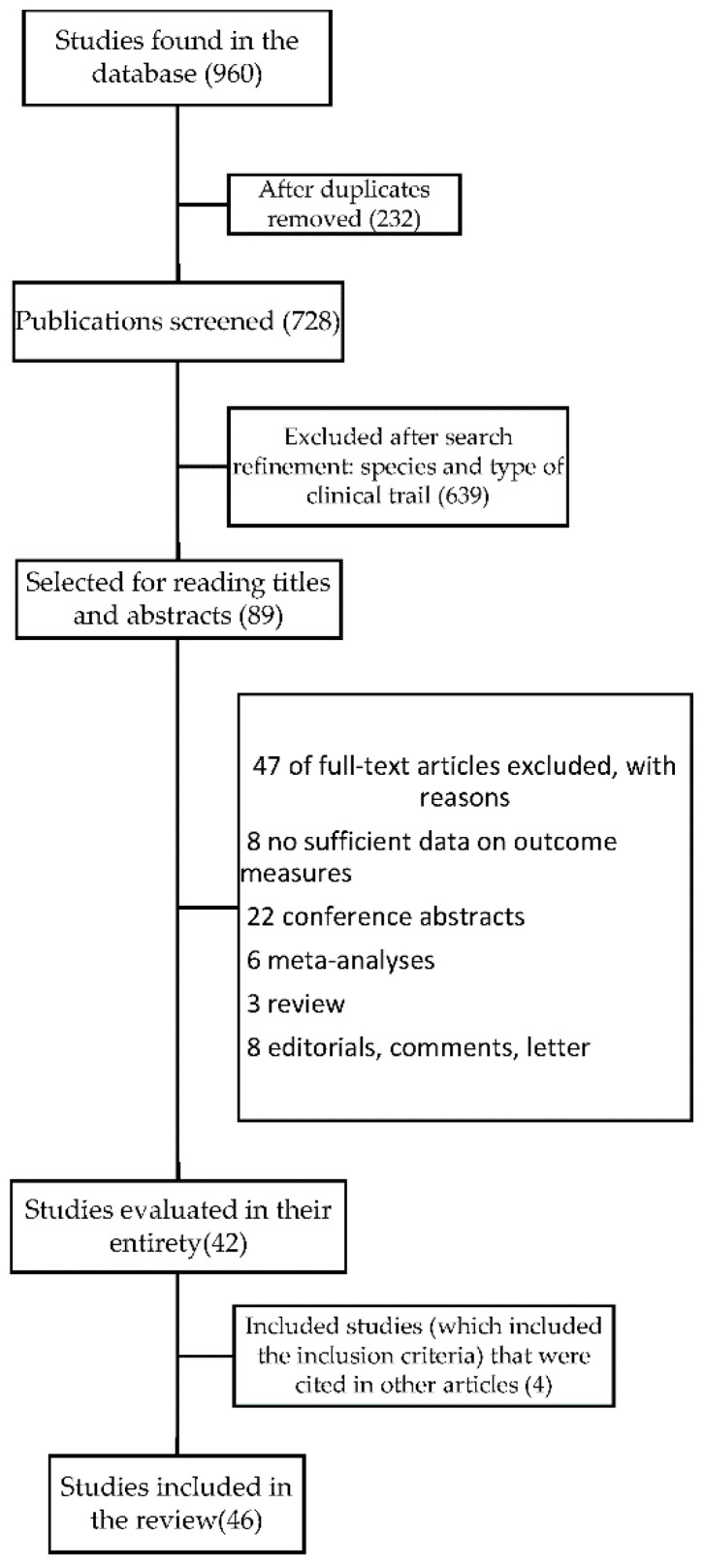
Steps for selecting the articles included in the review.

We found 960 articles, taking into account: clinical studies, human studies, and the last ten years. After the deletion of 232 duplicates, 639 articles were excluded by reviewing the titles and summaries, and the remaining 89 articles were reviewed to check the full text. Of the 89 studies, 46 were included in this review (Figure 1).

### 3.2. Risk of Bias Assessment

**Figure 2:**
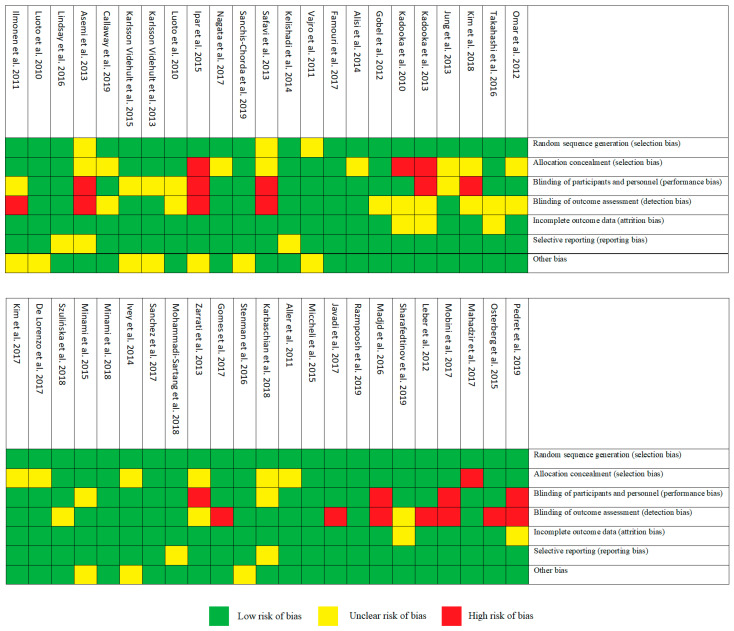
Risk of bias summary.

**Figure 3:**
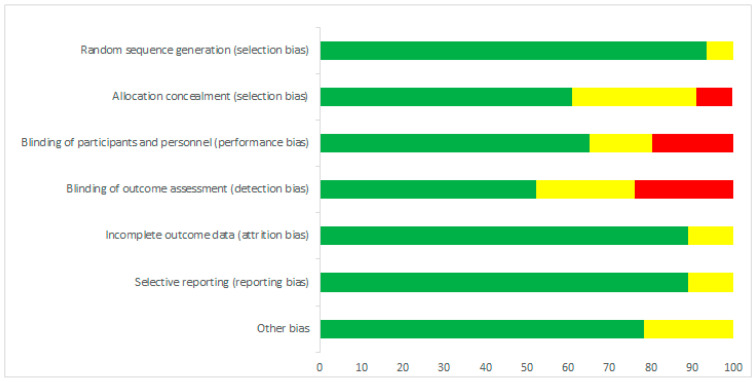
Risk of bias graph.

All selected articles have been evaluated for the risk of bias, as recommended by Cochrane [4]. Figure 2 and Figure 3 show the risk of bias according to the categories defined in the Cochrane tool. A low risk of bias was demonstrated in categories: random sequencing generation (93%), incomplete outcome data (89%) and selective reporting (89%). On the other hand, allocation concealment (30%) was characterized by a high percentage of unclear risk of bias. In the case of blinding of outcome assessment (24%) and blinding of participants and personnel (20%), we found high risks of bias.

## 4. Obesity

Obesity is the result of excessive energy supply to the body’s energy expenditure [5]. Excess energy is stored in the host’s fatty tissue, which leads to cell enlargement and impairment of their function [6]. Enlarged fat cells produce many biologically active substances called adipokines that act both locally (autocrine/paracrine) and on distant organs (hormonal activity) [7]. Adipokines have anti-inflammatory or proinflammatory effects. Adiponectin, C1q/TNF related proteins (CTRP), ominine, and soluble protein that inhibit the WNT 5 (SFRP5) signaling pathway belong to the anti-inflammatory adipokine family [8]. The proinflammatory adipokine family includes leptin, resistin, visfatin, retinol binding protein 4 (RBP4), lipocalin 2, IL-18, angiopoietin 2-like protein (ANGPTL2), chemokine ligand CC2 (CCL2), and ligand chemokine CXC 5 (CXCL5) [9]. An increase in these factors leads to the development of chronic inflammation and leads to a disruption of glucose metabolism and insulin resistance, which are the cause of type 2 diabetes [10]. Obesity also leads to a lipid profile disorder, an increase in blood pressure, and activation of procoagulative processes [11].

**Figure 4:**
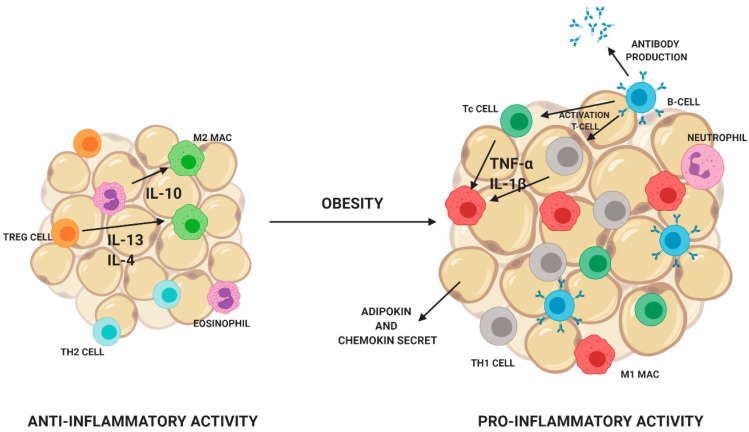
Inflammatory processes in the fat tissue. In obese people, the composition and number of cells in the immune system change. Reduction in the number of Treg lymphocytes, eosinophilia, and M2 macrophages and an increase in the number of B, Th1 lymphocytes, neutrophils, and M1 macrophages. Created with BioRender.com.

The secretion of adipocytokines depends on body fat composition [12]. In addition to adipocytes, adipose tissue consists of monocytes, lymphocytes, fibroblasts, macrophages, eosinophils, and vascular wall cells [13]. The number and distribution of cells in adipose tissue depend on its type and degree of obesity [14]. Fat tissue of lean people consists mainly of Treg lymphocytes, Th2 lymphocytes, eosinophils, and M2 macrophages. Eosinophils release anti-inflammatory cytokines IL-4 and IL13. Treg lymphocytes are responsible for the secretion of anti-inflammatory IL-10 and the stimulation of M2 macrophages to produce IL-10. People, who are overweight or obese, have an increase in the number of cells of the immune system in adipose tissue, which promotes the development of inflammation. In adipose tissue, a decrease in the number of eosinophils and a decrease in the diversity of T lymphocytes are visible. A decrease in the number of Treg lymphocytes and an increase in the number of proinflammatory CD8 + Tc and CD4 + Th1 cells. Monocytes are transformed into M1 proinflammatory macrophages. Additionally, an increase in the B lymphocyte count leads to an increase in the M1 macrophage count (Figure 4) [15,16].

### 4.1. The Role of Gut Microbiota in Obesity 

**Figure 5:**
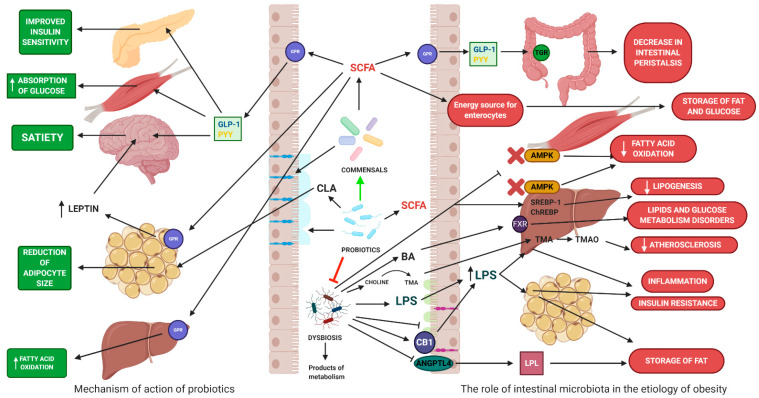
Mechanism of action of probiotics in the gastro intestinal tract: Probiotics increase the variety of intestinal microbiota. Inhibition of growth of pathogenic bacteria. Stimulating SCFA production in the gut. Regulation of appetite. Effects on glucose and lipid metabolism. Positive effect on intestinal barrier tightness. The role of intestinal microbiota in the etiology of obesity: Decreased AMPK expression causes a decrease in β-oxidation in the liver and muscles. Inhibition of ANGPTL4 increases lipoprotein lipase activity, which leads to TG storage in adipose tissue. Lowering GLP-1 levels disrupts the host’s response to insulin. Low levels of PYY cause more hunger. An increased hepatic lipogenesis by activating acetyl CoA carboxylase and fatty acid synthase. The development of inflammation in adipose tissue is caused by LPS. Stimulation of GPR 41/43 receptors increases lipolysis in adipose tissue. Activation of CB1 receptors. Abbreviations: SCFA—short chain fatty acids, GPR41/43—G-protein coupled receptor 41/43, GLP1—Glucagon Like Protein 1, PYY—peptide YY, LPS—lipopolysaccharide, TMA—trimethylamine, TMAO—Trimethylamine-N-oxide, TGR5—transmembrane G protein-coupled receptor, FXR—farnesoid X receptor, ANGPTL4—Angiopoietin Like 4, AMPK—5’AMP-activated protein kinase. Created with BioRender.com.

The intestines are colonized by over 1000 species of bacteria, mainly anaerobic, followed by facultative anaerobes and aerobes [17]. Dominant bacteria belong to these phyla: Bacteroidetes, Firmicutes, and Actinobacteria [18]. In a smaller amount, the intestines are colonized by Fusobacteria, Verrucomicrobia, Proteobacteria, and Cyanobacteria [18]. The intestinal microbiota also consists of archaea, viruses, and eukaryotes [18]. Bacteria that are part of the intestinal microbiota perform various functions, and their correct quantitative and qualitative structure supports the homeostasis of the whole organism [19]. However, this composition can change under the influence of many exogenous and endogenous factors [20]. Changes in the structure of microbiota caused by external factors may promote the development of metabolic diseases. However, the exact mechanisms linking changes in microbial composition and the development of obesity remain unclear due to the complex etiology of these diseases. GM is believed to contribute to obesity through chronic low-grade inflammation, excessive accumulation of lipids, metabolic disorders, and insulin resistance (Figure 5) [21,22,23].

### 4.2. The Impact on Energy Homeostasis

Intestinal microbiota can participate in obtaining energy via the fermentation of nondigestible dietary components in the large intestine [24]. This digestion is possible due to enzymes produced by bacteria [25]. Fermentation produces many compounds, of which short-chain fatty acids (SCFA) are the most important group, which include primarily acetic, propionic, and butyric acid [25]. The role of SCFA in obesity formation is not exactly known. On the one hand, an elevated SCFA level was found in obese individuals. SCFAs can be used by colonocytes, hepatocytes, and muscle cells as an energy source [25]. SCFA can account for about 10% of the host’s energy requirements [26]. Acetate and propionate play an important role in lipid and glucose metabolism. Acetate is used as a lipogenesis substrate in the liver, while propionate can be used in gluconeogenesis pathways. Excess SCFA is stored in the form of lipids and glucose. On the other hand, the influence of SCFA by GPR41 (FFAR2) and GPR43 (FFAR3) receptors and the tightness of intestinal membrane may have a positive effect on body weight. [26]. Propionic, acetic, and butyric acids are ligands for GPR41 (FFAR2) and GPR43 (FFAR3) receptors, which participate in the regulation of lipid and glucose metabolism [27]. SCFA can increase the oxidation of fatty acids in the liver and muscle tissue. Acetic and propionic acid stimulates adipocytes to synthesize leptin, a hormone with a strong anorectic effect [28]. SCFAs affect plasma glucose levels by increasing the secretion of intestinal hormones (YY peptide—PYY and glucagon-like peptide 1—GLP-1). The intestinal hormones affect the satiety center and reduce the appetite. In contrast, PYY and GLP-1 slow down the passage of nutrients in the intestines and thus increase the absorption of nutrients [29]. SCFAs have been shown to lower plasma cholesterol levels. Propionate reduces the activity of enzymes involved in the synthesis of cholesterol in the liver (synthase 3-hydroxy-3-methylglutaryl-CoA—HMGCS and reductase 3-hydroxy-3-methylglutaryl-CoA—HMGCR) [30]. In addition, SCFAs have an anti-inflammatory effect, reducing low-intensity chronic inflammation. SFCAs inhibit NF-κ B (nuclear factor κ-light chain-enhancer of activated B cells) activity, causing the reduction of the production of proinflammatory cytokines. SCFAs affect plasma LPS levels by regulating the integrity of the epithelial barrier, inducing the synthesis of tight junctions proteins and mucins [31] (Table 1).

Bile acids (BAs) are involved in cholesterol metabolism, fat digestion, and fat-soluble vitamin absorption [34]. In hepatocytes, cholesterol is converted to cholic acid (CA) and chenodeoxycholic acid (CDCA) classified as basic bile acids [35]. Binding of BAs to the farnesoid X receptor (FXR) inhibits the expression of sterol binding protein 1c (SREBP-1c), which regulates the synthesis of triglycerides (TG) in the liver by induction of key enzymes involved in lipogenesis such as fatty acid synthase (FAS). Activation of the FXR increases fatty acid oxidation (FFA) by increasing the expression of the receptor activated by the peroxisome α proliferator (PPARα) and reduces the production of very low-density lipids (VLDLs) in the liver. In addition, stimulation of FXR leads to reduction of gluconeogenesis in the liver, inhibition of glycolysis and induction of glycogen synthesis [35]. Activating FXR reduces plasma glucose levels and improves tissue sensitivity to insulin. Primary BAs in the large intestine are converted into secondary bile acids (deoxycholic and litholic acids) by the intestinal microflora. Secondary BAs are ligands of the G 5 protein-coupled receptor (TGR5), which has a beneficial effect on glucose homeostasis by stimulating glucagon-1-like peptide (GLP-1) expression and reducing TG levels in the liver. Intestinal microbiota, by changing the pool of BAs in the intestine, can affect the host’s body weight [36]. Mice without FXR (Fxr -/-) showed elevated cholesterol, TG, and excessive fat storage in the liver [37]. Administration of the FXR agonist (GW4064) to obese mice increased body weight and impaired glucose metabolism, leading to insulin resistance [38]. Improvement of metabolic parameters was achieved by administering FXR (glycine-β-murycholic acid) antagonists to obese mice [39]. Besides, BAs regulates the composition of the microbiota through a strong antibacterial effect. BAs affect phospholipids and membrane proteins, disrupting their functions [40].

Adenosine monophosphate kinase (AMPK) is an enzyme that is expressed mainly in the liver and skeletal muscles, and is involved in maintaining cellular energy homeostasis [41]. Phosphorylation of acetyl-CoA carboxylase (ACC) by AMPK reduces its activity and leads to a decrease in production of malonyl-CoA, an inhibitor of carnitine palmitoyltransferase (CPT1). Activation of CPT-1 increases the transport of fatty acids to the mitochondria where they are oxidized [42]. Gastrointestinal bacteria can inhibit AMPK activity [43], where it leads to a reduction in fatty acid oxidation, ketogenesis, and glucose uptake as well as increased cholesterol and TG synthesis [44]. In a study on mice bred under sterile conditions, higher AMPK levels were found in muscle and liver compared to mice bred under normal conditions [44]. These mice are more resistant to diet-induced obesity [45]. On the other hand, obese patients have increased liver TG production [46]. The increase in TG production is correlated with the increase in the level of fatty acid synthetase and acetyl CoA carboxylase [46]. The regulation of expression of these enzymes is associated with the action of transcription factors, such as SREBP-1c and the carbohydrate response protein (ChREBP) [47]. Increased fermentation of polysaccharides with the participation of intestinal microbiota increases the level of monosaccharides in the liver, leading to the activation of lipogenesis enzymes by ChREBP and SREBP-1 [48]. The resulting TGs are stored in adipose tissue and liver [49]. Choline is an essential component of the cell membrane [50]. It can be synthesized de novo or taken with food [50]. Low levels of choline in the liver lead to impaired VLDL formation and TG accumulation in the liver [46]. GM, through the metabolism of choline to trimethylamine, affects the bioavailability of choline and indirectly the storage of TG in the liver. In addition, the increased intestinal choline metabolism mediated by *Erysipelotrichia* spp. leads to a decrease in choline levels and an increase in the concentration of trimethylamine, which is converted to trimethylamine N-oxide (TMAO) in the liver [51]. TMAO affects lipid metabolism, resulting in cholesterol accumulation in cells and platelet activity leading to atherosclerosis [47]. Angiopoietin-like protein 4 (ANGPTL4)/fasting adipocytic factor (FIAF) is an active molecule produced by adipose tissue that is responsible for inhibiting the activity of lipoprotein lipase (LPL), responsible for the breakdown of TG in chylomicrons into fatty acids and glycerol [49]. The released compounds are transported to fat tissue and stored as fat [46]. In humans, the highest expression of ANGPTL4 is observed in the liver, adipose tissue, and small intestine [52]. Blocking the expression of ANGPTL4 by the intestinal microbiota leads to an increase in LPL activity, thus intensifying energy storage processes in the form of fat [53]. In the study of obese children, a correlation of body weight with ANGPTL4 was noted. Children with obesity had lower ANGPTL4 levels compared to healthy children. Besides, low ANGPTL levels correlated with lipid profile disorders. Weight reduction increased ANGPTL4 (70).

### 4.3. The Impact on Inflammatory Processes

The entry of pathogens into the body causes the development of an immune response, including an inflammatory response [54]. A short-lived inflammatory process leads to the elimination of the pathogen [55]. In contrast, chronic inflammation is a pathological condition that can damage host tissues [55]. Numerous scientific papers provide evidence confirming the relationship between the development of obesity and increased inflammatory activity within adipose tissue [56]. Lipopolysaccharides (LPS), also called endotoxins, are a component that builds the outer cell membrane of Gram-negative bacteria, which are responsible for initiating inflammatory processes leading to chronic generalized inflammation and endotoxemia [57]. Inflammation adversely affects metabolism. After reaching the general circulation, LPS triggers the immune response causing fatty liver and insulin resistance [58]. In healthy people, LPS passes into the bloodstream to a small extent [59]. A decrease in the synthesis of occludin and zonulin-1 proteins leads to a disruption of the integrity of the gastrointestinal mucosa [60]. A leaky intestinal barrier promotes the penetration of LPS through it [60]. Endotoxins form a complex with plasma LPS binding proteins (LBPs) [61]. The complex thus formed binds to the Toll-like 4 (TLR4) receptor on the surface of macrophages, which leads to the activation of NFκB and increasing the expression of genes responsible for the production of chemokines, cytokines, and proinflammatory enzymes [62]. Besides, changes in the composition of intestinal microbiota activate the endocannabinoid system by stimulating CB1 receptors, which consequently increases metabolic endotoxemia [63].

### 4.4. The Role of Probiotics in the Treatment and Prevention of Obesity

The mechanisms by which probiotics influence anthropometric parameters (body weight, waist circumference, and hip circumference) are not well known (Figure 3). It seems that the primary mechanism of action is related to changing the composition of intestinal microbiota. The use of probiotics modulates the intestinal microbiota by increasing the number of *Bifidobacterium* spp. and lactic acid sticks responsible for producing SCFA [64]. Probiotics influence appetite and energy homeostasis through increased SCFA production [65]. It has been shown that some *Bifidobacterium* spp. and *Lactobacillus* spp. produce prohealthy conjugated linoleic acid (CLA). CLA affects body weight by improving energy metabolism and lipolysis [66]. Additionally, the administered probiotics increase the amount of *Akkermansia muciniphila*, which has a positive effect on mucus thickness and intestinal barrier integrity. The beneficial effect is associated with a reduction in serum LPS levels and an improvement in the metabolic profile (reduction in total cholesterol, LDL, and TG levels in plasma and an increase in HDL cholesterol) [67]. In addition, probiotics have a beneficial effect on the populations of *Faecalibacterium prausnitzii*, an important buttermilk producer group. *F. prausnitzii* has an anti-inflammatory effect [68]. On the other hand, probiotics produce bacteriocin and organic acids, creating an unfavorable environment for the growth of opportunistic pathogens and their metabolites such as TMA, LPS, and indole [69]. Another possible mechanism of action of probiotics is related to the reduction of chronic systemic inflammation of low intensity occurring in obesity. Increased intestinal permeability leads to increased plasma LPS levels and increased expression of proinflammatory cytokines. Cytokines contribute to insulin resistance, oxidative stress, and increased visceral fat deposition. The administration of probiotics strengthens the intestinal barrier, increasing the production of tight junction proteins and mucins [70].

Probiotics contribute to reducing the size of adipocytes by decreasing the absorption of fatty acids and increasing the expression of genes associated with the oxidation of fatty acids [71]. *Lactobacillus rhamnosus* GG (LGG) inhibits fat accumulation in the liver by phosphorylation of AMPK [72]. *B breve B-3* ($10^{9}$ CFU per day) increased expression of ANGPTL4 in the intestines. ANGPTL4 can contribute to reduce fat accumulation in the fat tissue by inhibiting LPL [73]. *Lactobacillus* stimulates the production of certain cytokines, such as tumor necrosis factor alpha (TNF-α), and therefore can be effective in regulating leptin gene expression. In addition, the production of SCFA during the fermentation of prebiotic fiber can have a positive effect on adiponectin secretion. Leptin and adiponectin are strong anorexigenic hormones that inhibit food intake by the receptors present in the central nervous system [74].

The relationship between intestinal microbiota and body weight is very complex and further research is needed to clarify the role of probiotics in the prevention and treatment of obesity.

## 5. Review of Clinical Studies Using Probiotics in Obesity

Since 2004, when the composition of intestinal microbiota has been shown for the first time to have an influence on the energy production from nutrients, a great interest in the role of microbiota in the development of obesity has been observed. This discovery allowed us to develop a new concept of fighting overweight and obesity. The aim of this work was to present the current state of knowledge on the influence of doses, intervention time, and strains on body weight in humans (Table 2).

### 5.1. The Effect of Probiotic Supplementation in Pregnant Women on Body Weight

Women who are overweight or obese have an increased risk of pregnancy-induced hypertension, preeclampsia, and gestational diabetes (GDM). Research shows that supplementation with probiotics improves insulin sensitivity, reduces fasting glucose and insulin, and regulates glucose metabolism. Supplementation with *L. rhamnosus* GG and *B. lactis* ($10^{10}$ per day) from the first-trimester pregnancy up to 6 months after delivery in combination with a diet can counteract obesity and reduce metabolic disorders [75]. Additionally, the study showed long-term benefits and safety associated with the intervention used, among others reducing the risk of GDM in women at high risk [76,123]. On the other hand, there was no effect of *L. salivarius* UCC118 ($10^{9}$ CFU per day) on body weight during four-week supplementation in women in the first trimester of pregnancy [77]. Similar results were obtained using 200 g of yogurt contained *S. thermophilus, L. bulgaricus, L. acidophilus* LA5, and *B. animalis* BB12 strains ($10^{7}$ CFU/g per day) for 9 weeks [78]. Additionally, probiotic supplementation (*L. rhamnosus* and *B. animalis* ssp. *lactis* $10^{9}$ CFU) in overweight or obese women in the second trimester of pregnancy did not prevent GDM [79]. The presented results show unclear results of the effect of probiotic supplementation in pregnant women with GDM. A dose above $10^{7}$ CFU may show a beneficial effect on the metabolic profile of pregnant women. Several limitations of studies should be noted. The study involved small groups of women, from 50 to 250 people. The duration of the study and the observation period for the effects of the actions of probiotics was too short. Additionally, the study involved women in various trimesters of pregnancy, which makes interpretation of the results difficult. Therefore, further research with longer intervention and more participants is needed.

### 5.2. The Effect of Probiotic Supplementation in Infants in the Prevention of Overweight or Obesity

A small number of studies assessing the long-term effect of probiotic supplementation on the prevention of overweight and obesity have been conducted. Karlsson et al. [80,81] evaluated the effect of supplementation of porridge containing *L. paracasei* ($10^{8}$ CFU per day for six months) strain in four-month-old infants. BMI, body mass, metabolic, and inflammatory markers did not differ between the control group and the probiotic group in children aged eight to nine years. In another randomized clinical trial, Luoto et al. [82] evaluated the effect of probiotic supplementation in pregnant women on the development of obesity in their offspring. The women received *L. rhamnosus GG* ($10^{10}$ CFU per day) from 4 weeks before expected delivery. The intervention is extended by 6 months after the birth. The children were observed for up to 10 years after birth. Perinatal probiotic intervention alleviates excessive weight gain, especially in children who were later overweight in the first years of life (the most visible at the age of 4). The presented works constitute a new concept of combating obesity by modifying the composition of intestinal microbiota during infancy. Further studies with more infants are necessary to confirm these results.

### 5.3. The Effect of Probiotic Supplementation in Children on Body Weight

Obesity in childhood and adolescence can lead to the development of heart disease and type 2 diabetes. In several clinical studies, the influence of probiotic supplementation in children on body weight and body mass index (BMI) was checked. In children receiving probiotics, a statistically significant weight reduction was noted. Administration of the synbiotic (*L. acidophilus, L. rhamnosus, B. bifidum, B. longum*—each 4.3 × $10^{8}$ CFU/sachet, *E. faecium* 8.2 × $10^{8}\;\mathrm{CFU}$/s, fructooligosaccharides (FOS), lactulose, vit. A, B1, B2, B6, E, C per day) for 4 weeks in obese children significantly reduces body weight and BMI [83]. Nagata et al. [84] compared the effect of using fermented milk (FM) (*L. casei* Shirota $4\times 10^{10}\;\mathrm{CFU}$ per day) with a dietary intervention combined with physical activity. After 6 months of FM supplementation, a decrease in body weight, and an increase in plasma HDL levels were observed. A 6-month dietary treatment combined with physical activity did not have a significant effect on weight loss. Supplementation of *B. pseudocatenulatum* CECT 7765 (1 capsule $10^{9}$–$10^{10}$ CFU per day) for 13 weeks reduced the BMI in obese children with insulin resistance [85]. In a clinical trial involving obese children and adolescents, supplementation with a mixture of probiotics (*L. casei, L. rhamnosus, St. thermophilus, B. breve, L. acidophilus, B. longum,* and *L. bulgaricus* $2\times 10^{8}$ CFU per day) with prebiotic (vitamin A, C, and E) significantly reduced body weight, BMI and waist circumference compared to the control group [86,87]. Additionally, the studies showed beneficial effects of probiotics on other companion diseases of obesity. Among other things, a decrease in inflammation (decrease in the level of inflammatory markers—IL-6 and TNFα), insulin resistance, and cardioprotective effect through the influence on lipid profile (decrease in TG and LDL levels and increase in HDL levels) were noted.

Non-alcoholic fatty liver disease (NAFLD) is currently one of the leading causes of chronic liver disease in children. The increase in obesity and overweight in children results in a higher incidence of NAFLD in this group. Current medical interventions and lifestyles offer little effectiveness in treating NAFLD in children, and other therapeutic interventions are not approved for children. A study on the effect of *L. rhamnosus* GG supplementation on obese children with non-alcoholic steatosis has shown improvement in liver function. USG examination showed a decrease in the liver with a parallel reduction in ALT and AST liver enzyme activity. No weight reduction and BMI were observed in the study [88]. In a triple-blind clinical trial (*L. acidophilus ATCC* B3208 3 × $10^{9}$ CFU; *B. lactis* DSMZ 32269 6 × $10^{9}$ CFU, *B. bifidum* ATCC SD6576, and *L. rhamnosus* DSMZ 21690 each 2 × $10^{9}$ CFU 1 capsule per day for 12 weeks), Famouri et al. [89] achieved improved liver function and weight reduction. Additionally, the probiotics used improved lipid profile in obese children with NAFLD. Alisi et al. [90] conducted a double-blind clinical trial in obese children with NAFLD and demonstrated that a 4-month VSL #3 supplement significantly improves BMI and liver functions in obese children. In a randomized, double-blind, placebo-controlled clinical trial, Gobelt et al. [91] checked the effect of *L. salivarius* Ls-33 supplementation ($10^{10}$ CFU per day for 12 weeks) in 50 obese teenagers. After 12 weeks, compared to the placebo group, they found no difference in anthropometric measurements, glucose and insulin levels on fasting, lipid profile (TC, HDL, LDL, TG, and free fatty acids), blood pressure, interleukin (IL)-6, and TNF-α.

These studies show the potential effect of probiotics on body weight and BMI in obese or overweight children. Modulation of intestinal microbiota with probiotics can be a tool to alleviate some obesity-related disorders in children. Additionally, the conducted studies provide evidence of the safety of short term use of probiotics in children. Unfortunately, a significant limitation of the studies is the small size of study groups and the short duration of the studies and observations. In most clinical trials, strain mixtures were used. To better understand the effect of probiotics on body weight, studies with single probiotics should be designed in the future.

### 5.4. The Effect of Probiotic Supplementation in Adults on Body Weight

The influence of probiotics on body weight was best studied in adults. The beneficial effect of weight reduction was obtained during supplementation with *L. gasseri* in people with overweight and obesity. Daily consumption of 200 g of yoghurt containing *L. gasseri* SBT2055 ($10^{8}$ CFU/g per day) for 12 weeks significantly reduced BMI, waist and hip circumference, and body fat mass. Positive intervention-related changes diminished 4 weeks after the end of probiotic supplementation, indicating that continuous intake of *L. gasseri* SBT2055, even at a low dose ($10^{6}$ CFU per g milk), is necessary to reduce obesity-related consequences [92,93]. Similar effects were achieved with high ($10^{10}$ CFU) and low ($10^{9}$ CFU) doses of *L. gasseri* BNR17 daily for 12 weeks [94,95]. Consumption of yoghurt containing *B. lactis* ssp. *lactis* GCL2505 for 12 weeks reduces visceral fat. Visceral obesity is one of the factors leading to metabolic disorders. Supplementation of *B. animalis* ssp. *lactis* GCL2505 can be used to reduce abdominal obesity in overweight people [96]. Similar results were obtained during consumption of 100 g of yoghurt containing *L. amylovorus* (1.39 × $10^{9}$ CFU) or *L. fermentum* (1.08 × $10^{9}$ CFU) for 6 weeks leads to a decrease in total body fat mass. The effect was stronger during the supplementation of *L. amylovorus* [97]. In overweight individuals, supplementation with a probiotic preparation containing *L. plantarum* KY1032 and *L. curvatus* 8HY7601 for 12 weeks resulted in a significant decrease in body weight and subcutaneous fatty tissue [98] A similar effect was obtained using a probiotic mixture (*B. bifidum* SGB02, *B. animalis* subsp. *lactis* SGB06, *S. thermophilus* SGSt01, *S. thermophiles, L. plantarum* SGL07, *L. delbrueckii* spp. *bulgaricus* DSM 20081, *L. reuteri* SGL01, *L. acidophilus* SGL11, and *Lactococcus lactis* subsp. *lactis* SGLc01) for three weeks [99]. High-dose supplementation (1 × $10^{10}$ CFU) of preparations containing several probiotic strains in postmenopausal women (*B. bifidum* W23, *L. salivarius* W24, *L. acidophilus* W37, *B. lactis* W51, *B. lactis* W52, *L. casei* W56, *L. brevis* W63, *Lactococcus lactis* W19, and *L. lactis* W58) for 12 weeks caused a decrease in glucose, lipopolysaccharides, total cholesterol, and insulin. Besides, the tested dose reduced waist circumference and fat tissue mass [100]. Regular supplementation of *B. brevi* B-3 (2 × $10^{10}$ CFU 2 or 3 capsules per day for 12 weeks) reduces fat mass and has a positive effect on liver function **[101,102]**. On the other hand, the supplementation of *L. acidophilus* La5 and *B. animalis* subsp. *lactis* Bb12 (3 × $10^{9}$ CFU per day for 6 weeks) in combination with the diet did not affect the body weight and metabolic markers [103].

Interesting observations were noted in a randomized, double-blind, placebo-controlled clinical trial, Sánchez et al. [104] showed differences in the effects of synbiotic between genders. After twelve-week supplementation of synbiotic (*L. rhamnosus* CGMCC1.3724 (3.24 × $10^{8}$) resulted in more significant weight loss in obese women than in men. The consumption of synbiotic affects the intestinal-brain axis, reducing the feeling of hunger.

Several clinical studies have compared the use of probiotics with diet or prebiotics. The study suggests that regular consumption of fortified yoghurt containing *S. thermophiles*, *L. bulgaricus,* and enriched $10^{7}$ CFU/g of *B. lactis* Bb-12, inulin, whey protein, vitamin D3, and calcium helps to reduce body weight and improve metabolic status in obese individuals [105,106]. The isocaloric diet combined with probiotic supplementation (*B. lactis, B. bifidum, L. casei, L. acidophilus,* and *Lactococcus lactis* each $10^{9}$ CFU per day) for eight weeks significantly reduced the level of polyunsaturated fatty acids and waist circumference in obese or overweight women. These results suggest that the supplementation of the probiotic mix and diet is more effective than diet [107]. Similar results were obtained use of *B. lactis* ssp. *lactis 420* ($10^{10}$ CFU per day) with or without the addition of fiber for six months. This allowed one to reduce fat mass in overweight and obese adults [108].

In addition, the use of probiotics and prebiotics may increase the effectiveness of current obesity treatment methods. Supplementation of synbiotic containing *L. casei* (3.5 × $10^{9}$ CFU)*, L. rhamnosus* (7.5 × $10^{8}$ CFU), *L. bulgaricus* ($10^{8}$ CFU), *L. acidophilus* ($10^{9}$ CFU), *B. breve* ($10^{10}$ CFU)*, B longum* (3.5 × $10^{9}$ CFU), *S. thermophilus* ($10^{8}$ CFU), and FOS for 16 weeks (from 4 weeks before surgery to 12 weeks after surgery) in patients after gastric bariatric surgery compared to placebo significantly improved anthropometric parameters [109].

Overweight and obesity lead to the development of metabolic diseases such as type 2 diabetes, insulin resistance, or non-alcoholic fatty liver disease. In patients with metabolic diseases, quantitative and qualitative disorders of intestinal microbiota occur. In several clinical trials, the influence of probiotic supplementation on insulin resistance, lipid profile, glucose level, and markers of the inflammatory state in obese individuals has been tested. Supplementation with *Lactobacillus* spp. and *Bifidobacterium* spp. in adults with non-alcoholic fatty liver disease (NAFLD) improved liver function. Patients had a decreased level of alanine aminotransferase and aspartate aminotransferase, an increase in GLP-1 and decrease liver steatosis. Supplementation with probiotics reduces TNF-α and oxidative stress markers. In addition, the improvement in liver function was accompanied by a reduction in BMI [110,111,112,124]. The use of a mixture of probiotics *Lactobacillus* spp., *Bifidobacterium* spp., and *Streptococcus thermophilus* (VSL#3) caused a significant decrease in total cholesterol (TC), low-density lipids (LDLs), very low-density lipids (VLDLs), TG, and C-reactive protein after six weeks of supplementation. The mixture of probiotics used improved insulin sensitivity and increased the level of high-density lipids (HDL) [125]. The use of a supplement consisting of seven live strains of *Lactobacillus* spp., *Bifidobacterium* spp., and *Streptococcus* spp. ($10^{8}-10^{10}$ CFU twice a day for 6 weeks) caused a significant decrease in fasting plasma glucose, BMI, and an increase in HDL-C concentration in patients with type 2 diabetes [113]. On the other hand, consumption of yogurt enriched with *B. lactis* BB12 and *L. acidophilus* LA5 strains for 12 weeks significantly reduced total cholesterol, LDL, and insulin resistance, but did not affect body weight [114]. A beneficial effect on body weight and morning blood pressure was given when yogurt enriched with *L. plantarum* (1.5 × $10^{11}$ 50 g per day for 3 weeks) [115]. In contrast, the supplementation with *L. casei* Shirota (6.5 × $10^{9}$ three times a day for 12 weeks) strain did not affect inflammation markers and body mass in patients with metabolic syndrome. The lack of effect is associated with too short a study duration or probiotic overdose [116]. A similar effect was achieved during supplementation of *L. reuteri* DSM 17938 ($10^{8}\;\mathrm{or}\;10^{10}$ CFU per day) for 12 weeks did not affect HbA1c, liver steatosis, and obesity [117,118].

Finally, healthy people without being overweight or obese can also benefit from the use of probiotics. The study assessed the effect of VSL#3 on high-fat diet-induced obesity (HFD). Healthy adults were fed HFD (55% fat, 30% carbohydrates, and 15% protein) and VSL #3 (4.5 × $10^{10}$ CFU daily) for 4 weeks. The results showed that VSL#3 supplementation prevents excess weight development [119].

### 5.5. The Effect of Heat Killed Probiotics on Body Weight

The safety profile of live probiotics is not precisely defined. The main risks during the use of probiotics concern systemic infections caused by translocation, especially in sensitive patients (immunodeficiency) and children or the acquisition of antibiotic resistance genes. To avoid this risk, there is a growing interest in non-viable microorganisms, mainly thermally killed. In a double-blind clinical study Pedret et al. have determined the effect of *B. animalis* subsp. *lactis* CECT 8145 (Ba8145) ($10^{10}$ CFU per day for 12 weeks) in the form of live cells and subjected to high temperature on body weight. In obese individuals, the ingestion of Ba8145, both the live and modified form, improves anthropometric parameters [120]. Similar effects in the form of weight reduction, waist circumference, and BMI were obtained using *Pedicoccus pentosaceus* ($10^{11}$ CFU per day for 12 weeks) [121]. Despite promising results, more studies are needed. In subsequent tests, the safety of heat-killed probiotics should be determined. In addition, it is necessary to identify which heat-killed probiotics may be promising candidates for the prevention or treatment of disease and to carry out tests with more individuals.

### 5.6. The Effect of Probiotic Supplementation on Weight Gain

Probiotic supplementation may also cause weight gain. Jones et al. [122] conducted trial in 19 obese adolescents, administering three packets per day of a mixture probiotics (*L. acidophilus* BA05, *L. plantarum* BP06, *L. paracasei* BP07, *L. delbrueckii* subsp. *bulgaricus* BD08, *B. breve* BB02, *B. longum* BL03, *B. infantis* BI04, and *S. thermophilus* BT01) for 16 weeks. Compared to placebo, observed a statistically significant increase in body weight in people using VLS#3. Weight gain may be associated with non-compliance with diet by the subjects. The results obtained need to be confirmed in subsequent studies on more people.

## 6. Meta-Analyses

Meta-analyses conducted in recent years provide ambiguous results on the influence of probiotics in the prevention or treatment of overweight and obesity. A meta-analysis conducted by Park et al. [126] (four RCT studies) did not show a significant effect of probiotics on body weight and BMI. However, the meta-analysis carried out has severe limitations. The works analyzed differ in the duration of the intervention, the doses used, and the strains. Besides, only four RCTs were used in the meta-analysis. Similar results were obtained in a review (nine studies) with 410 adolescents and children (duration of intervention 4–16 weeks) [127]. The use of probiotic/synbiotic supplements did not have a beneficial effect on the weight of children and adolescents. The lack of effect is probably due to the high heterogeneity of research. Different probiotic strains were used in the analyzed studies, each of which may have different effects on the microbiota. This is important because only certain strains of probiotics can regulate body weight. On the other hand, a meta-analysis based on 15 clinical trials involving 957 people (intervention time was 3–12 weeks) resulted in a significant reduction in BMI and body weight compared to placebo. However, the effect achieved was small [128]. Similar results were obtained in a meta-analysis conducted by Zhang et al. [129] on a group of 1931 people over 18 years of age. Based on 25 studies, it was found that the consumption of probiotics could significantly reduce body weight and BMI. The best effect was achieved in the population of overweight and obese people, as the administration of probiotics lasted longer than eight weeks. The study also suggests a better impact of multi-strain probiotics. The meta-analysis of Koutnikov et al. [130] (6826 patients) also showed an improvement in anthropometric parameters during probiotic supplementation. The review confirmed the beneficial effect of preparations consisting of three or more strains. Similar results were obtained in a meta-analysis based on 12 randomized controlled trials (821 participants) [66]. Interesting results were obtained in the review involving adults, children and infants. 2–3 months of probiotic use in adults resulted in a significant but small weight loss. A small weight gain in children was observed during the use of *Lactobacillus* spp. for 8–26 weeks. Among infants, consumption of preparations enriched in probiotics from 3 weeks to 10 months was associated with a significant increase in body weight [131].

## 7. Conclusions

A diet rich in fats, preservatives, and carbohydrates, and low in fiber, typical of developed countries, harms the composition of the intestinal microbiota. Besides, these changes are compounded by stress and the use of certain drugs, including antibiotics, proton pump inhibitors, and nonsteroidal anti-inflammatory drugs. Quantitative, qualitative, and functional disorders in the intestinal microbiota cause the development of inflammation, which leads to metabolic disorders such as obesity or diabetes. Probiotic supplementation has beneficial effects on both anthropometric and metabolic parameters.

However, before probiotics can be used to treat obesity and other metabolic diseases, some very important questions need to be answered. First, determine which groups of bacteria are involved in the etiology of obesity. In some studies, the strains had a positive effect, while in other studies the opposite results were obtained. The role of SCFA in the formation of obesity should also be determined. On the one hand, SCFA supplementation has a beneficial effect on body weight in overweight or obese individuals. On the other hand, many studies have shown an increased level of SCFA in fecal samples of obese individuals as compared to lean individuals. It is unclear if the beneficial effect of SCFAs is weakened in obese individuals or if the effect is not strong enough to offset the adverse effects of diet. It is also important to determine effective doses and the maximum duration of the use of probiotics. Besides, subsequent studies need to determine the impact and safety of long-term use of probiotics on human health.

In summary, the intake of probiotics can have a modulating effect on body weight and BMI. Weight reduction was greater among the population treated for longer. Research also suggests a stronger effect of the mix of probiotic strains than individual bacterial species. Additionally, weight reduction was intensified by using probiotic preparations with prebiotics (symbiotic), diet, and physical activity. The presented research presents the benefits of modifying the composition of the intestinal microbiota as a promising strategy for the treatment of obesity.

## Figures and Tables

**Table 1:** Properties of short chain fatty acids [32,33].

Short-Chain Fatty Acid (SCFA)	Receptors	Properties
Acetic acid	GPR 41, GPR43	Stimulates the secretion of intestinal hormones PYY and GLP-1—appetite reduction Increased production of leptin Precursors for cholesterol and fat acids synthesis
Propionic acid	GPR 41, GPR43	Stimulates the secretion of intestinal hormones PYY and GLP-1—appetite reduction Inhibition of fat accumulation in adipocytes Increased production of leptin Substrate of gluconeogenesis
Butyric acid	GPR 41, GPR43, GPR109A	Differentiation of Treg-cell Decrease the production of IL-12 Increase the production of IL-10 Stimulation of fat storage in adipose tissue Promotes intestinal epithelial integrity Increase in the oxidation of fatty acids Stimulating production of mucin

**Table 2:** Impact of probiotic supplementation on obesity and overweight.

Author	Study Design	Duration of Intervention	Intervention	Sample (Age)	Control/Placebo Groups	Clinical Outcome (vs. Control/Placebo Group)	Reference
Ilmonen et al.	Randomized placebo-controlled trial	From the first trimester, pregnancy up to 6 months after delivery	*L. rhamnosus GG, B. lactis* ($10^{10}$ CFU) 1 capsule per day	256 pregnant women; 85 diet/probiotics; 86 diet/placebo; 85 control/placebo (25–35 years)	1 capsule containing microcrystalline cellulose and dextrose per day/without diet	↓WC (*p* < 0.001)	[75]
Luoto et al.	Randomized, double-blind, placebo-controlled trial	From the first trimester to the end of breastfeeding	*L. rhamnosus GG, B. lactis* ($10^{10}$ CFU) 1 capsule per day	256 pregnant women; 85 diet/probiotics; 86; diet/placebo; 85 control/placebo	1 capsule containing microcrystalline cellulose and dextrose per day/without diet	Reduction in gestational diabetes (GDM) (13% diet/probiotics; 36% diet/placebo; 34% control/placebo) (*p* < 0.003)	[76]
Lindsay et al.	Randomized, double-blind, placebo-controlled trial	4 weeks	*L. salivarius UCC118* ($10^{9}$ CFU) 1 capsule per day	138 women in the first trimester of pregnancy; 63 probiotic group; 75 placebo group (>18 years)	1 capsule per day	No effect	[77]
Asemi et al.	Randomized single-blinded controlled trial	9 weeks	200 g of yogurt containing *L. acidophilus LA5* and *B. animalis BB12* ($10^{7}$ CFU/g) per day	67 women in the third trimester of pregnancy; 33 probiotic group; 37 placebo group (18–30 years)	200 g of conventional yoghurt per day	No effect	[78]
Callaway et al.	Prospective double-blind RCT	From 20 weeks gestation to delivery	*L. rhamnosus, B. animalis* subspecies *lactis* ($10^{9}$ CFU) per day	211 obese and overweight women; 207 probiotic; 204 placebo (>18 years)	1 capsule containing microcrystalline cellulose and dextrose per day	No effect	[79]
Karlsson Videhult et al.	Randomized, double-blind, placebo-controlled trial	From 4 to 13 months of age	Cereals containing of *L. paracasei* ssp. *paracasei*—LF19 ($10^{8}$ CFU) per day	120 children; 58 probiotic group; 62 placebo group (8–9 years)	Cereals without LF19	Effect on anthropometric parameters (*p* < 0.05)	[80]
Karlsson Videhult et al.	Randomized, double-blind, placebo-controlled trial	From 4 to 13 months of age	Cereals containing of *L. paracasei* ssp. *paracasei*—LF19 ($10^{8}$ CFU) per day	120 children; 58 intervention group; 62 placebo group (8–9 years)	Cereals without LF19	No effect	[81]
Luoto et al.	Randomized, double-blind, prospective follow-up	Mothers 4 weeks before delivery; infants for 6 months after birth	*L. rhamnosus GG* ($10^{10}$ CFU) 1 capsule per day	113 Mother-child; 54 probiotic group; 59 placebo group	1 capsule containing microcrystalline cellulose per day	Probiotic administration may reduce weight gain, especially up to 4 years of age (*p* = 0.08)	[82]
Ipar et al.	Open-label, randomized, controlled trial	4 weeks	*L. acidophilus, L. rhamnosus, B. bifidum, B. longum* (each 4.3 × $10^{8}$ CFU), *E. faecium* (8.2 × $10^{8}$ CFU), FOS 625mg, lactulose 400 mg, vit. A 6 mg, B1 1.8 mg, B2 1.6 mg, B6 2.4 mg, E 30 mg, C 75 mg 1 sachet per day	117 children with primary obesity; 77 intervention group + calorie reduction + physical activity; 40 calorie reduction + physical activity (5–17 years)	Calorie reduction + physical activity	↓BMI (*p* < 0.05); ↓ HC and WC (*p* < 0.05)	[83]
Nagata et al.	Open-label prospective	6 months	1 bottle of fermented milk contained *L. casei* Shirota (4 × $10^{10}$) per day	12 obese children; 22 healthy non-obese children	Diet + physical activity	↓ weight (*p* < 0.05)	[84]
Sanchis-Chorda et al.	Open-label prospective	13 weeks	*B. pseudocatenulatum* CECT 7765 ($10^{9}$$–10^{10}$ CFU) 1 capsule per day	48 obese children with insulin resistance; 24 probiotic + diet; 24 diet (10–15 years)	1 capsule per day	Improved lipid profile (*p* = 0.035), inflammatory markers (*p* = 0.026) and ↓ BMI (*p* = 0.001)	[85]
Safavi et al.	Randomized triple-blinded controlled trial	8 weeks	*L. casei, L. rhamnosus, S. thermophilus, B. breve, L. acidophilus, B. longum* and *L. bulgaricus* (Each 2 × $10^{8}$ CFU), vit. E, A, C per day	56 obese children and adolescents; 29 intervention group; 27 placebo (6–18 years)	1 capsule containing maltodextrin per day	↓ BMI (*p* = 0.002), ↓ WC (*p* = 0.001)	[86]
Kelishadi et al.	Triple-blinded controlled RTC	8 weeks	*L. casei, L. rhamnosus, S. thermophilus, B. breve, L. acidophilus, B. longum* and *L. bulgaricus* (Each 2 × $10^{8}$ CFU), vit. E, A, C per day	56 obese children and adolescents; 29 probiotic; 27 placebo (6–18 years)	1 capsule containing maltodextrin per day	↓ BMI (*p* = 0.002)	[87]
Vajro et al.	Double-blind placebo-controlled pilot study	8 weeks	*L. rhamnosus* GG (12 billion CFU/day)	20 obese children with persisting hypertransaminasemia and ultrasonographic bright liver; 10 probiotic; 10 placebo (10–13 years)	Placebo	No effect	[88]
Famouri et al.	Randomized triple-blind placebo-controlled trial	12 weeks	*L. acidophilus ATCC B3208* (3 × $10^{9}$ CFU), *B. lactis DSMZ 32269* (6 × $10^{9}$ CFU), *B. bifidum ATCC SD6576, L. rhamnosus DSMZ 21690* (each 2 × $10^{9}$ CFU) 1 capsule per day	64 obese children and adults with NAFLD; 32 probiotics group; 32 placebo group (10–18 years)	1 capsule per day	Improvement of lipid profile (*p* < 0.001) and NAFLD (*p* < 0.05); ↓ plasma liver enzymes (*p* = 0.02); No effect on BMI and body weight	[89]
Alisi et al.	Parallel double-blind RCT	4 months	Age < 10 year 1 sachet per day of VSL#3, age > 10 year 2 sachets of VSL#3	44 children with NAFLD; 22 probiotic group; 22 placebo group (9–12 years)	Age < 10 year 1 sachet per day, age > 10 year 2 sachets of VSL#3	↓ BMI (*p* = 0.001); ↑ GLP-1 (*p* = 0.001)	[90]
Gobel et al.	Randomized double-blind placebo-controlled trial	12 weeks	*L. salivarius LS 33* ($10^{10}$ CFU) 1 capsule per day	50 obese adolescents; 27 probiotic; 23 placebo (12–15 years)	1 capsule containing cellulose, silicon dioxide and rice-maltodextrin per day	No effect	[91]
Kadooka et al.	Multi-centre, double-blind, parallel group RCT	12 weeks	200 g of fermented milk containing *L. gasseri SBT2055* ($10^{8}$ CFU/g) per day)	87 obese adults; 43 probiotic group, 44 placebo group (33–63 years)	200 g of fermented milk without probiotic per day	↓ Weight, BMI, HC and WC (*p* = 0.01)	[92]
Kadooka et al.	Multi-centre, double-blind, parallel group RCT	12 weeks	200 g of fermented milk containing *L. gasseri SBT2055* ($10^{6}$ or $10^{7}$ CFU/g) per day	210 obese adults; 69 high dose probiotic, 71 low dose probiotic, 70 placebo (35–60 years)	200 g of fermented milk without probiotic per day	↓BMI, HC, WC and body fat mass (*p* = 0.01)	[93]
Jung et al.	Randomized, double-blind, placebo-controlled trial	12 weeks	*L. gasseri BNR17* ($10^{10}$ CFU) 6 capsules per day	57 overweight or obese adults; 28 probiotic group; 29 placebo group (19–60 years)	6 capsules containing trehalose, skim milk, and fructooligosaccharide per day	↓ WC and HC (*p* = 0.015); ↓ body weight (*p* = 0.008)	[94]
Kim et al.	Randomized, double-blind, placebo-controlled trial	12 weeks	*L. gasseri BNR17* ($\mathrm{low}\;10^{9}\mathrm{or}\;\mathrm{high}\;10^{10}$ CFU) 2 capsuls per day	90 overweight or obese adults; 30 high dose probiotic; 30 low dose probiotic; 30 placebo (20–75 years)	2 capsule containing maltodextrin, crystalline cellulose, and magnesium stearate per day	High and low probiotic doses reduce body weight and waist circumferences (*p* < 0.05); A high dose of probiotic reduces visceral adipose tissue (*p* < 0.05)	[95]
Takahashi et al.	Multicenter, randomized, double-blind, placebo-controlled intervention trial	12 weeks	100g of fermented milk containing *B. animalis* ssp. *lactis* GCL2505 (8 × $10^{10}$ CFU) per day	137 overweight and obese adults; 69 probiotic; 68 placebo (20–65 years)	100 g of fermented milk without probiotic	↓ visceral fat (*p* = 0.05)	[96]
Omar et al.	Randomized double-blind placebo-controlled trial	43 days	100 g of yogurt containing *L. amylovorus* (1.39 × $10^{9}$ CFU) or 100 g of yogurt containing L. fermentum (1.08 ×$10^{9}$ CFU) per day	28 overweight and obese adults; 14 probiotic LA; 12 probiotic LF; 12 placebo (16–60 years)	100 g of control yogurt per day	No effect	[97]
Kim et al.	Randomized, double-blind, placebo-controlled trial	12 weeks	2 g of powder *containing L. plantarum KY1032* and *L. curvatus* (2.5 × $10^{9}$ CFU) twice a day	65 overweight adults; 32 probiotic; 34 placebo (25–75 years)	2 g of powder without probiotic twice a day	↓ body weight (*p* < 0.05)	[98]
De Lorenzo et al.	Randomized, double-blinded controlled trial	3 weeks	*S. thermophilus SGSt01, B. animalis* subsp. *Lactis SGB06, S. thermophiles, B. bifidum SGB02, L. delbrueckii* spp. *Bulgaricus DSM 20081, L. lactis* subsp. *Lactis SGLc01, L. acidophilus SGL11, L. plantarum SGL07, L. reuteri SGL01* (1.5 × $10^{10}$ CFU) 1 sachet per day	48 women with normal weight, overweight or obesity; 24 probiotics; 24 placebo (20–65 years)	1 sachet containing maltodextrin from corn and silica per day	↓ BMI, and fat mass (*p* < 0.05)	[99]
Szulińska et al.	Randomized, double-blind, placebo-controlled trial	12 weeks	*2* g of powder containing *B. bifidum W23, B. lactis W51, B. lactis W52, L. acidophilus W37, L. brevis W63, L. casei W56, L. salivarius W24, L. lactis W19,* and *L. lactis* *W58* (High dose $10^{10}$ CFU or low dose 2.5 × $10^{9}$ CFU per day divided in two equal doses)	71 obese women; 23 high dose probiotic; 24 low dose probiotic; 24 placebo (45–70 years)	2 g of powder containing only the excipients, i.e., maize starch and maltodextrins	Improving lipid profile and glucose metabolism (*p* < 0.05); ↓ visceral fat, waist circumference (*p* < 0.05); High probiotic doses ↓ LPS levels (*p* < 0.05)	[100]
Minami et al.	Randomized, double-blind, parallel-group comparative trial	12 weeks	*B. breve B-3* (5 × $10^{10}$ CFU, 3 capsules per day)	44 overweight adults with diabetes; 19 probiotic group; 25 placebo group (40–69 years)	3 capsules per day containing maize starch	Improving liver function (*p* < 0.05); Reduction of metabolic disorders (*p* < 0.05); Reduction of fat mass (*p* < 0.05)	[101]
Minami et al.	Randomized, double-blind, placebo-controlled trial	12 weeks	*B. breve B-3* (2 × $10^{10}$ CFU) 2 capsuls per day	80 healthy pre-obese adults; 40 probiotics; 40 placebo (20–64 years)	2 capsules per day containing corn starch	↓ body fat (*p* < 0.05); No effect on lipid profile	[102]
Ivey et al.	Randomized double-blinded parallel study	6 weeks	Both the probiotic yogurt and the probiotic capsule contained *L. acidophilus La5, B. animalis* subsp. *lactis Bb12* (3 × $10^{9}$ CFU per day)	156 overweight men and women; 40 probiotic yoghurt + probiotic capsules; 37 probiotic yoghurt + placebo capsules; 39 control milk + probiotic capsules; 40 control milk + placebo capsules (over 55 years)	Control milk	No effect	[103]
Sanchez et al.	Randomized, double-blind, placebo-controlled trial	12 weeks	*L. rhamnosus CGMCC1.3724* (3.24 × $10^{8}$ CFU) + 90 g inulin and 210 g FOS, 2 capsules per day	125 obese adults; 62 intervention group, 63 placebo group (18-55 years)	2 capsules containing maltodextrin and magnesium stearate per day	↓ weight (*p* = 0.02); ↓ appetite (*p* = 0.02)	[104]
Mohammadi-Sartang et al.	Randomized double-blinded controlled trial	10 weeks	250 g of fortified yogurt (FSY) with whey protein, calcium, vit. D, fiber and probiotic (*S. thermophiles, L. bulgaricus* and at least $10^{7}\mathrm{CFU}/g$ *B. lactis Bb-12*) 250 g twice a day	87 obese adults with metabolic syndrome; 44 fortified yogurt + diet; 43 low-fat conventional yogurt + diet (20–65 years)	250 g of low fat plain yogurt with *St. thermophiles* and *L. bulgaricus* (LFY) twice a day	↓ body fat mass (*p* < 0.05); ↓ WC (*p* < 0.05), ↓ TG ↓ HOMAR-IR value (*p* < 0.05); ↑HDL (*p* < 0.05)	[105]
Zarrati et al.	Randomized double-blind placebo-controlled trial	8 weeks	200 g of yogurt containing *L. acidophilus La5, B. BB12* and *L. casei DN001* $10^{8}$ CFU/g	75 obese adults; 25 regular yogurt + low calorie diet; 25 probiotic yogurt + low calorie diet; 25 probiotic yogurt without low calorie diet	200 g of yogurt without probiotics	↓ body mass and inflammation (*p* < 0.001)	[106]
Gomes et al.	Randomized, double-blind, placebo-controlled trial	8 weeks	*L. acidophilus LA-14, L. casei LC-11, L. lactis LL-23, B. bifidum BB-06, B. lactis BL-4* (each $10^{9}$ CFU) 4 sachets per day + diet	43 overweight or obese women; 21 probiotics; 22 placebo (20–59 years)	4 sachets per day of placebo + diet	↓ abdominal adiposity (*p* = 0.02)	[107]
Stenman et al.	Randomized, double-blind, placebo-controlled, multi-center clinical trial	6 months	*B. animalis* ssp. *lactis 420* ($10^{10}$ CFU) with or without 12g of LU per day	134 overweight or obese adults; 25 probiotic; 36 LU (12 g); 37 probiotic + LU (12 g); 36 placebo (18–65 years)	1 sachet (12 g) containing microcrystalline cellulose per day	↓ fat mass and WC (*p* = 0.02)	[108]
Karbaschian et al.	Randomized, double-blind, placebo-controlled trial	4 weeks before surgery and 12 weeks after surgery	*L. casei* (3.5 × $10^{9}$ CFU/g), *L. rhamnosus* (7.5 ×$10^{8}$ CFU/g), *S. thermophilus* ($10^{8}$ CFU/g), *B. breve* ($10^{10}$ CFU/g), *L. acidophilus* ($10^{9}$ CFU/g), *B. longum* (3.5 × $10^{9}$ CFU/g), and *L. bulgaricus* ($10^{8}$ CFU/g) and 38.5 mg FOS 1 capsule per day	46 Obese women; 23 probiotics; 23 placebo (18–60 years)	1 capsule containing maltodextrin per day	↓ body weight (*p* < 0.05)	[109]
Aller et al.	Randomized, double-blind, parallel-group comparative trial	3 months	*L. bulgaricus* and *St. thermophilus* (500 million of probiotic, 1 tablet per day)	28 adults with NAFLD; 14 probiotic group; 14 placebo group (39–60 years)	1 tablet contained 120 mg of starch	Improving glucose metabolism (*p* < 0.05); Improvement of liver function (*p* < 0.05); No effect on body weight	[110]
Miccheli et al.	Parallel-arm, double-blind RCT	4 months	(112.5 ×$10^{9}$ CFU) - VSL#3 (age < 10 year 1 sachet per day of VSL#3; age > 10 year 2 sachets of VSL#3) + diet + physical activity	31 children with NAFLD; 15 probiotic group; 16 placebo (9–12 years)	1 sachet placebo	↓ BMI (*p* = 0.045) and liver function (*p* = 0.026)	[111]
Javadi et al.	Randomized double-blind placebo-controlled trial	3 months	*B. longum, L. acidophilus* (each 2 × $10^{7}$ CFU per day)	75 obese adults: 20 probiotic; 19 prebiotic; 17 probiotic + prebiotic; 19 placebo (20–60 years)		Supplementation with probiotic and/or prebiotic improves liver function (*p* < 0.05); ↓ body mass and BMI (*p* < 0.05)	[112]
Razmpoosh	Randomized, double-blind, placebo-controlled trial	6 weeks	*L. acidophilus* (2 × $10^{9}$ CFU), *L. casei, B. longum* (each 7 ×$10^{9}$ CFU), *L. rhamnosus* (1.5 × $10^{9}$ CFU), *L. bulgaricus* (2 × $10^{8}$ CFU), *B. breve* (3 × $10^{10}$ CFU), *S. thermophilus* (1.5 × $10^{9}$ CFU), 100 mg FOS 2 capsules per day	60 adults with diabetes; 30 probiotic group, 30 placebo group (30–75 years)	2 capsules per day containing fructooligosaccharide and magnesium stearate	↓ fasting glucose (*p* = 0.001); ↑ HDL (*p* = 0.002); No effect on anthropometric parameters and insulin levels	[113]
Madjd et al.	Randomized, single-blind, controlled trial	12 weeks	200 g of probiotic yogurt (*S. thermophiles, L. bulgaricus, L. acidophilus LA5, B. lactis BB12* - $10^{7}$ CFU) twice a day	89 healthy overweight and obese women; 45 probiotic; 44 placebo (18–50 years)	200 g low-fat yogurt twice a day	No effect	[114]
Sharafedtinov et al.	Randomized, double-blind, placebo-controlled trial	3 weeks	50 g cheese with *L. plantarum TENSIA* $\left(1.5\times 10^{11}\;\mathrm{CFU}/g\right)$ per day	40 obese adults with hypertension; 25 probiotic group; 15 placebo group (30–69 years)	50 g cheese without probiotic per day	↓ BMI and morning blood pressure (*p* = 0.001)	[115]
Leber et al.	Open label, randomized pilot study	3 months	1 bottles (65 mL) containing *L. casei Shirota* (6.5 × $10^{9}$ CFU) three times a day	28 adults with MetS; 13 probiotic; 15 placebo (24–66 years)	1 bottles (65 mL) of placebo three times a day	No effect	[116]
Mobini et al.	Randomized double-blind placebo-controlled trial	12 weeks	Powder containing *L. reuteri DSM 17938* low dose ($10^{8}$ CFU) or high dose ($10^{10}$ CFU)	44 patients with type 2 diabetes; 15 low dose; 14 high dose; 15 placebo (50–75 years)	Powder without probiotic	No effect	[117]
Mahadzir et al.	Randomized triple-blind placebo-controlled trial	4 weeks	*L. acidophilus BCMC 12130, L. casei* subsp. *BCMC 12313, L. lactis BCMC 12451, B. bifidum BCMC 02290, B. longum BCMC 02120* and *B. infantis BCMC 02129* (3 × $10^{10}$ CFU per day) twice a day	24 overweight adults; 12 probiotics; 12 placebo (18–50 years)	2 sachets without probiotic	No effect	[118]
Osterberg et al.	Randomized, double-blind, parallel-group comparative trial	4 weeks	VLS#3 (4.5 × $10^{10}$) 2 sachets per day	20 healthy non-obese; 9 probiotic; 11 placebo group (18–30 years)	2 sachets per day	↓ body mass (*p* = 0.023)	[119]
Pedret et al.	Randomized, parallel, double-blind, placebo-controlled trial	12 weeks	*B. animalis* subsp. *lactis CECT 8145* ($10^{10}$ CFU) 1 capsul per day; heat-killed *B. animalis* subsp. *lactis CECT 8145* ($10^{10}$ CFU) 1 capsule per day	126 abdominally obese adults, 42 probiotic; 44 heat killed from probiotic; 40 placebo group (>18 years)	1 capsule containing 300 mg of maltodextrose	↓ BMI and WC (*p* < 0.05)	[120]
Higashikawa et al.	Randomized double-blind placebo-controlled trial	12 weeks	Powder containing living *Pediococcus pentosaceus LP28* ($10^{11}$ CFU) with dextrin per day; Powder containing heat-kill *Pediococcus pentosaceus LP28* ($10^{11}$ CFU) with dextrin per day	62 overweight adults; 21 living LP28; 21 heat-killed LP28; 20 placebo group (20–70 years)	Powder containing dextrin	↓ body mass (*p* = 0.004), BMI (*p* = 0.035), WC (*p* = 0.009) in group after Heat-killed LP28	[121]
Jones et al.	Randomized double-blind placebo-controlled trial	16 weeks	VLS#3 3 sachets per day	19 obese adolescents; 8 probiotic; 11 placebo group (12–18 years)	3 sachets per day	↑ total adiposity (*p* = 0.01)	[122]

VLS#3—*B. longum, B. infantis, B. breve, L. acidophilus, L. paracasei, L. delbrueckii* subsp. *bulgaricus*, and *L. plantarum, S. salivarius* subsp. *thermophilus*; LU—The dietary fiber Litesse Ultra polydextrose. FOS—fructooligosaccharides. HC—hip circumferences. WC—waist circumference.
